# Bacterial synthesis of *N*-hydroxycinnamoyl phenethylamines and tyramines

**DOI:** 10.1186/s12934-015-0353-y

**Published:** 2015-10-13

**Authors:** Geun Young Sim, So-Mi Yang, Bong Gyu Kim, Joong-Hoon Ahn

**Affiliations:** Department of Bioscience and Biotechnology, Bio/Molecular Informatics Center, Konkuk University, Seoul, 143-701 Republic of Korea; Department of Forest Resources, Gyeongnam National University of Science and Technology, 33 Dongjin-ro, Jinju-si, Gyeongsangman-do 660-758 Republic of Korea

**Keywords:** Hydroxycinnamate, Hydroxycinnamate amine, Metabolic engineering

## Abstract

**Background:**

Hydroxycinnamic acids (HCAs) including cinnamic acid, *p*-coumaric acid, caffeic acid, and ferulic acid, are C6–C3 phenolic compounds that are synthesized via the phenylpropanoid pathway. HCAs serve as precursors for the synthesis of lignins, flavonoids, anthocyanins, stilbenes and other phenolic compounds. HCAs can also be conjugated with diverse compounds including quinic acid, hydroxyl acids, and amines. Hydroxycinnamoyl (HC) amine conjugates such as *N*-HC tyramines and *N*-HC phenethylamines have been considered as potential starting materials to develop antiviral and anticancer drugs.

**Results:**

We synthesized *N*-HC tyramines and *N*-HC phenethylamines using three different approaches in *Escherichia coli*. Five *N*-HC phenethylamines and eight *N*-HC tyramines were synthesized by feeding HCAs and phenethylamine or tyramine to *E. coli* harboring *4CL* (encoding 4-coumarate CoA:ligase) and either *SHT* (encoding phenethylamine *N*-HC transferase) or *THT* (encoding tyramine *N*-HC transferase). Also, *N*-(*p*-coumaroyl) phenethylamine and *N*-(*p*-coumaroyl) tyramine were synthesized from *p*-coumaric acid using *E. coli* harboring an additional gene, *PDC* (encoding phenylalanine decarboxylase) or *TDC* (encoding tyrosine decarboxylase). Finally, we synthesized *N*-(*p*-coumaroyl) phenethylamine and *N*-(*p*-coumaroyl) tyramine from glucose by reconstructing the metabolic pathways for their synthesis in *E. coli*. Productivity was maximized by optimizing the cell concentration and incubation temperature.

**Conclusions:**

We reconstructed the metabolic pathways for synthesis of *N*-HC tyramines and *N*-HC phenethylamines by expressing several genes including *4CL*, *TST or SHT*, *PDC* or *TDC*, and *TAL* (encoding tyrosine ammonia lyase) and engineering the shikimate metabolic pathway to increase endogenous tyrosine concentration in *E. coli*. Approximately 101.9 mg/L *N*-(*p*-coumaroyl) phenethylamine and 495.4 mg/L *N*-(*p*-coumaroyl) tyramine were synthesized from *p*-coumaric acid. Furthermore, 152.5 mg/L *N*-(*p*-coumaroyl) phenethylamine and 94.7 mg/L *N*-(*p*-coumaroyl) tyramine were synthesized from glucose.

## Background

Plants synthesize phenolic compounds through the phenylpropanoid pathway. Hydroxycinnamic acids (HCAs) serve as precursors for the synthesis of lignins, flavonoids, anthocyanins, stilbenes and other phenolic compounds [1]. Hydroxycinnamates can be conjugated with diverse compounds. In the formation of hydroxycinnamate conjugates, hydroxycinnamates such as cinnamic acid, *p*-coumaric acid, caffeic acid, and ferulic acid can serve as donors, while diverse chemicals including quinic acid, hydroxyl acids (maleic acid and tartaric acid), amino compounds (aromatic amino acids, choline, and anthranilic acids), and polysaccharides (glycerol, anthocyanin glycosides, flavonoid glycosides, and terpene glycosides) can be used as acceptors [2].

Hydroxycinnamoyl (HC) amides are an example of hydroxycinnamate conjugates, in which tyramine, phenethylamine, serotonin, anthranilate, tryptamine, or dopamine serve as HC acceptors. HC amides are formed in plants for defense against pathogens [3]. For example, *N*-cinnamoyl phenylethylamine was reported to show antifungal activity [4]. Accumulation of *N*-cinnamoyl tyramine and *N*-feruloyl tyramine in tomato (*Lycopersicon eseulentum*) was found to lead to resistance against *Pseudomonas syringae* [5]. Also, some plants synthesize *N*-HC phenethylamine and *N*-HC tyramine. *N*-(*p*-Coumaroyl) tyramine and *N*-feruoyl tyramine are found in tomato [6], *Solanum melongena* [7], and *Caspicum annuum* [8]. *N*-(*p*-Coumaoyl) phenethylamine has been isolated from *Anomianthus dulcis* [9].

Many plant metabolites have been used as starting molecules to develop new medicines [10, 11]. HC amides were originally known as antioxidants, like other plant phenolic compounds. However, recent studies have shown that HC amides could be developed into new candidate medicines. For example, hydroxycinnamate amide served as a backbone for the synthesis of antiviral compounds [12]. Analogs of *N*-HC phenalkylamides are inhibitors of tyrosinase in human melanocytes, which has the potential to treat pigmentation-related disorders [13]. *N*-HC tyramine showed anti-proliferative effects on cancer cells [14] and selectively induced the apoptosis of cancer cells [15].

For the synthesis of *N*-HC phenethylamines and *N*-HC tyramines, the two amino acids phenylalanine and tyrosine, respectively, serve as the key substrates. Phenethylamine and tyramine are synthesized from phenylalanine and tyrosine by phenylalanine decarboxylase (PDC) and tyrosine decarboxylase (TDC), respectively. *PDC* has been isolated and characterized from tomato [16] and TDC has been isolated from several plants including parsley, *Papaver somniferum*, *Arabidopsis thaliana*, and *Catharanthus roseus* [17–21]. PDC and TDC show catalytic activity for both phenylalanine and tyrosine as substrates, but have been named depending on their substrate preference. On the other hand, hydroxycinnamic acids are also synthesized from these two amino acids by phenylalanine ammonia lyase (PAL) and tyrosine ammonia lyase (TAL) via the phenylpropanoid pathway [22]. Therefore, the endogenous concentrations of tyrosine and phenylalanine are critical factors for the synthesis of *N*-HC tyramines and *N*-HC phenethylamines in *Escherichia coli*.

**Fig. 1:**
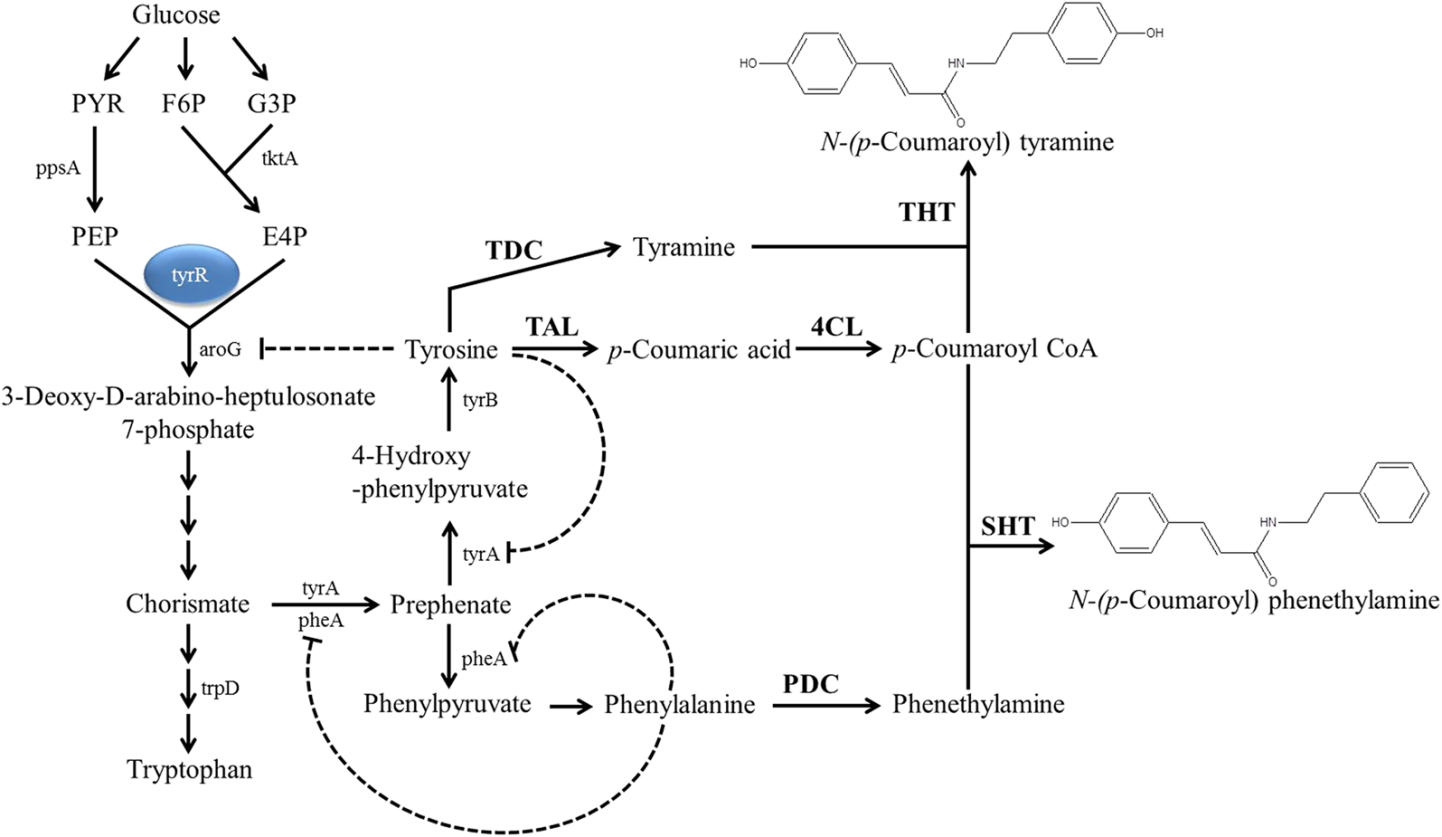
A schematic pathway for the synthesis of *N*-(*p*-coumaroyl) phenethylamine and *N*-(*p*-coumaroyl) tyramine in *Escherichia coli*. *ppsA* phosphoenolpyruvate synthetase, *tktA* transketolase, *tyrR* phenylalanine DNA-binding transcription repressor, *aroG* deoxyphosphoheptonate aldolase, *tyrA* prephenate dehydrogenase, *pheA* prephenate dehydratase, *tyrB* phenylalanine aminotransferase, *TAL* tyrosine amino lyase, *4CL* 4-coumaroyl-CoA ligase, *TDC* tyrosine decarboxylase, *PDC* phenylalanine decarboxylase, *THT* tyramine *N*-hydroxycinnamoyl transferase, *SHT* phenethylamine *N*-hydroxycinnamoyl transferase. Tyrosine inhibits tyrR and tyrA while phenylalanine inhibits pheA. *PDC*, *TDC*, *TAL*, *SHT*, and *THT* were introduced into *E. coli* to synthesize *N*-(*p*-coumaroyl) phenethylamine and *N*-(*p*-coumaroyl) tyramine

The formation of conjugates between HCs and phenethylamine or tyramine can be mediated by HC transferases (HCTs). HCTs use HC-CoA as the HC donor and various compounds including phenethylamine and tyramine as HC acceptors [23]. HC-CoA: tyramine *N*-(HC) transferase (THT) catalyzes the formation of *N*-HC tyramines; the corresponding gene has been cloned from *Capsicum annuum* [24]. HC-CoA:serotonin *N*-(HC) transferase (SHT) from *C. annuum* was shown to catalyze the formation of *N*-HC phenethylamines although it demonstrated higher affinity for serotonin than for phenethylamine [25]. Using these genes, it was possible to reconstruct the pathways for the synthesis of *N*-(*p*-coumaroyl) phenethylamine and *N*-(*p*-coumaroyl) tyramine in *E. coli* (Fig. 1).

In this report, we synthesized *N*-HC tyramines and *N*-HC phenethylamines from tyrosine and phenylalanine using metabolically engineered *E. coli*. The shikimate pathway of *E. coli* which leads to the production of phenylalanine, tyrosine, and tryptophan, is well-characterized and strategies to increase the intracellular concentrations of tyrosine and phenylalanine are also known. [26]. Using engineered *E. coli* strains, we were able to synthesize *N*-HC phenylethylamines and *N*-HC tyramines from glucose as well as from HCs.

## Results

### Production of *N*-HC phenethylamines and *N*-HC tyramines in *E. coli*

**Fig. 2:**
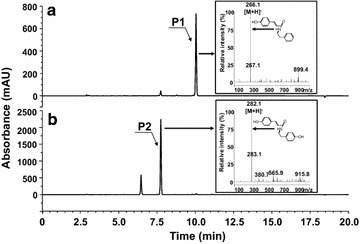
HPLC analysis of *N*-(*p*-coumaroyl) phenethylamine (**a**) and *N*-(*p*-coumaroyl) tyramine (**b**). *P1* and *P2* indicate the reaction products. The *inset* shows the mass spectra of the reaction products. The peak detected at 6.5 min in Fig. 1b is a *p*-coumaric acid

*N*-HC phenethylamines and *N*-HC tyramines were synthesized from HC and phenethylamine or tyramine. Two enzymatic reactions were required for the synthesis of these compounds. The first reaction was activation of HCA by attaching CoA to HCA to form HC-CoA, which is catalyzed by 4-coumarate CoA:ligase (4CL), and the second reaction was formation of the conjugate between HC-CoA and tyramine or phenethylamine, which is mediated by HCT. We cloned two genes, *4CL* from *Oryza sativa* and *SHT* from *C. annuum*, into an *E. coli* expression vector for the synthesis of *N*-HC phenethylamine. The resulting *E. coli* strain was named HP-1. For the synthesis of *N*-HC tyramines, we used strain HT-1, which expresses *4CL* from *O. sativa* and *THT* from *C. annuum*. The formation of the reaction products after feeding strain HP-1 or strain HT-1 with *p*-coumaric acid and phenethylamine or tyramine is shown in Fig. 2. Peaks of UV absorbance (P1 in Fig. 2a and P2 in Fig. 2b) obtained by high-pressure liquid chromatography (HPLC) were confirmed as *N*-(*p*-coumaroyl) phenethylamine and *N*-(*p*-coumaroyl) tyramine by mass spectrometry (data not shown). In addition, the structures of *N*-(*p*-coumaroyl) phenethylamine and *N*-*(p*-coumaroyl) tyramine were determined using nuclear magnetic resonance spectroscopy (NMR) (see “Methods”). We also observed the formation of a small amount of *N*-*(p*-coumaroyl) tyramine (peak at 7.8 min in Fig. 2a) during the formation of *N*-(*p*-coumaroyl) phenethylamine. Formation of *N*-(*p*-coumaroyl) phenethylamine was also observed during the synthesis of *N*-*(p*-coumaroyl) tyramine (peak at 10.1 min in Fig. 2b). An explanation for this is that yeast extract used in the growth medium contained phenethylamine and tyramine which might have served as substrates.

Twelve HCAs were tested to determine the substrate range of 4CL, SHT and THT using *E. coli* strains HP-1 and HT-1 (Table 1). Each HCA was fed to HP-1 along with phenethylamine. The strain HP-1 produced reaction products with five of HCs. Among them, 3-methoxy cinnamic acid was the best substrate, followed by caffeic acid, cinnamic acid, *p*-coumaric acid, and ferulic acid. For the formation of *N*-HC tyramines, strain HT-1 was used. Eight of twelve HCAs formed conjugates with tyramine. Again, 3-methoxy cinnamic acid was the best substrate, followed by ferulic acid, *m*-coumaric acid, cinnamic acid, caffeic acid, *o*-coumaric acid, *p*-coumaric acid, and 4-methoxy-cinnamic acid (Table 2). Among five naturally occurring HCAs (cinnamic acid, *p*-coumaric acid, caffeic acid, ferulic acid, and sinapic acid), all of them except sinapic acid led to the formation of either *N*-HC phenethylamines or *N*-HC tyramines. These results indicate that the size of each HC was a determining factor in whether they could act as substrates for the formation of the corresponding *N*-HC phenethylamines or *N*-HC tyramines. When cinnamic acids containing more than two methoxy groups were used as substrates, neither *N*-HC phenethylamines nor *N*-HC tyramines were formed.

**Table 1:** Plasmids and strains used in the present study

Plasmids or *E. coli* strain	Relevant properties or genetic marker(s)	Source or references
Plasmids		
pACYCDuet	P15A ori, Cm^r^	Novagen
pCDFDuet	CloDE13 ori, Str^r^	Novagen
pETDuet	f1 ori, Amp^r^	Novagen
pA-SeTAL	pACYCDuet carrying *TAL* from *Saccharothrix espanaensis*	[31]
pA-aroG-SeTAL-tyrA	pACYCDuet carrying *TAL* from *S. espanaensis*, *aroG* and *tyrA* from *Escherichia coli*	[31]
pA-aorG^fbr^-SeTAL-tyrA^fbr^	pACYCDuet carrying *TAL* from *S. espanaensis*, *aroG* ^fbr^, and *tyrA* ^fbr^ from *E. coli*	
pE-PDC	pETDuet carrying *PDC* from *Pseudomonas putida*	
pC-Os4CL-SHT	pCDFDuet carrying *4CL* from *Oryza sativa* and *SHT* from *Capsicum annuum*	
pC-Os4CL-THT	pCDFDuet carrying *4CL* from *O. sativa* and *THT* from *C. annuum*	This study
pC-TDC-Os4CL-THT	pCDFDuet carrying *TDC* from *Papaver somniferum*, *4CL* from *O. sativa*, and *THT* from *C. annuum*	This study
Strains		
BL21 (DE3)	F^−^ *ompT* *hsdS*_*B*_(r_B_^−^ m_B_^−^) *gal* *dcm* *lon* (DE3)	Novagen
BtyrR	BL21(DE3) *ΔtyrR*	[31]
BtyrR-trpD	BL21(DE3) *ΔtyrR/ΔtrpD*	This study
HP-1	BL21 harboring pC-Os4CL-SHT	This study
HP-2	BL21 harboring pC-Os4CL-SHT and pE-PDC	This study
HP-3	BL21 harboring pC-Os4CL-SHT, pA-SeTAL, and pE-PDC	This study
HP-4	BL21 harboring pC-Os4CL-SHT, pA-aroG-SeTAL-tyrA, and pE-PDC	This study
HP-5	BL21 harboring pC-Os4CL-SHT, pA-aroG^fbr^-SeTAL-tyrA^fbr^, and pE-PDC	This study
HP-6	BtyrR harboring pC-Os4CL-SHT, pA-aroG^fbr^-SeTAL-tyrA^fbr^, and pE-PDC	This study
HP-7	BtyrR-trpD harboring pC-Os4CL-SHT, pA-aroG^fbr^-SeTAL-tyrA^fbr^, and pE-PDC	This study
HT-1	BL21 harboring pC-Os4CL-THT	This study
HT-2	BL21 harboring pC-TDC-Os4CL-THT	This study
HT-3	BL21 harboring pC-TDC-Os4CL-THT and pA-aroG-tyrA	This study
HT-4	BL21 harboring pC-TDC-Os4CL-THT and pA-aroG^fbr^-tyrA^fbr^	This study
HT-5	BL21 harboring pC-TDC-Os4CL-THT and pA-SeTAL	This study
HT-6	BL21 harboring pC-TDC-Os4CL-THT and pA-aroG-SeTAL-tyrA	This study
HT-7	BL21 harboring pC-TDC-Os4CL-THT and pA-aroG^fbr^-SeTAL-tyrA^fbr^	This study
HT-8	BtyrR-pheA harboring pC-TDC-Os4CL-THT and pA-aroG^fbr^-SeTAL-tyrA^fbr^	This study
HT-9	BtyrR-trpD harboring pC-TDC-Os4CL-THT and pA-aroG^fbr^-SeTAL-tyrA^fbr^	This study

**Table 2:**
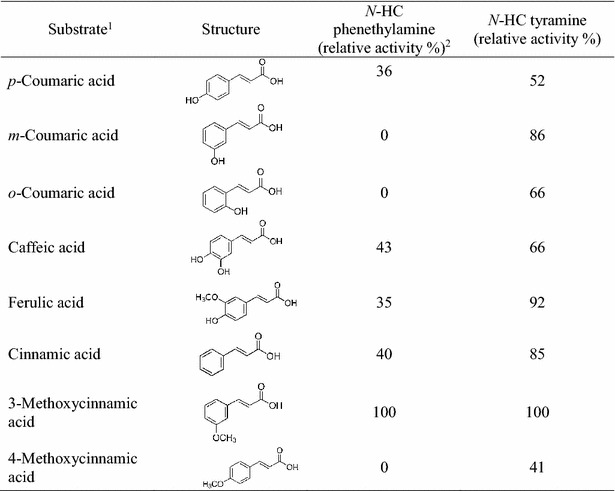
Relative conversion rates of various HCs using *E. coli* strains HP-1 and HT-1

^1^3,4-dimethoxycinnamic acid, 2,4-dimethoxycinnamic acid, sinapic acid, and 3,4,5-trimethoxycinnamic acid were tested but did not serve as substrates for either enzyme

^2^Relative activity was calculated by denoting the activity of the best substrate as 100 %

### Synthesis of *N*-(*p*-coumaroyl) phenethylamine in *E. coli*

Among the several reaction products, we decided to synthesize *N*-(*p*-coumaroyl) phenethylamine and *N*-(*p*-coumaroyl) tyramine by feeding *E. coli* with only *p*-coumaric acid. For this, either a decarboxylase that can convert phenylalanine into phenethylamine or one that can convert tyrosine into tyramine was introduced into the *E. coli* strains HP-1 and HT-1. We engineered strain HP-2 to synthesize *N*-(*p*-coumaroyl) phenethylamine by transforming *E. coli* with *PDC* along with *SHT* and *4CL* and then feeding them *p*-coumaric acid. Analysis of the cultures from strain HP-2 using HPLC showed a peak with the same retention time as that of *N*-(*p*-coumaroyl) phenethylamine and its structure as that compound was confirmed by MS and NMR (data not shown). Therefore, we decided to use the HP-2 strain for optimization of the production of *N*-(*p*-coumaroyl) phenethylamine. First, we tested the effect of initial cell density. The cell density of HP-2 was adjusted to an OD_600_ of 1, 3, 5, or 7. The production of *N*-(*p*-coumaroyl) phenethylamine increased from 22.8 mg/L at OD_600_ = 1, 55.7 mg/L at OD_600_ = 3, and 59.6 mg/L at OD_600_ = 5, but decreased to 51.6 mg/L at OD_600_ = 7. The optimal incubation temperature was determined at the optimized cell density of OD_600_ = 5. HP-2 grown at 25, 30, or 37 °C produced 43.1, 59.6, or 48.2 mg/L *N*-(*p*-coumaroyl) phenethylamine, respectively. The initial *p*-coumaric acid concentration was then optimized with the optimized cell density (OD_600_ = 5) and incubation temperature (30 °C). The strain HP-2 was fed with 0.2, 0.4, 0.6, 0.8, or 1.0 mM of *p*-coumaric acid and the production of *N*-(*p*-coumaroyl) phenethylamine was examined. HP-2 converted 81.8 % of 0.2 mM *p*-coumaric acid into *N*-(*p*-coumaroyl) phenethylamine, producing 43.7 mg/L *N*-(*p*-coumaroyl) phenethylamine. When the cells were fed with 0.4 mM *p*-coumaric acid, the conversion rate was 80.4 %, producing 85.9 mg/L *N*-(*p*-coumaroyl) phenethylamine. At higher *p*-coumaric acid concentrations, the conversion rate of *p*-coumaric acid was less than 50 % and the final yields of *N*-(*p*-coumaroyl) phenethylamine were 78.5, 74.0 and 59.6 mg/L, respectively, which were much lower than the yield at 0.4 mM *p*-coumaric acid. Using the optimized initial concentration of *p*-coumaric acid (0.4 mM), we monitored the production of *N*-(*p*-coumaroyl) phenethylamine and found that approximately 101.9 mg/L *N*-(*p*-coumaroyl) phenethylamine was produced after 12 h, after which the yield decreased until 24 h (data not shown). Approximately 95.3 % of *p*-coumaric acid was converted into *N*-(*p*-coumaroyl) phenethylamine.

**Fig. 3:**
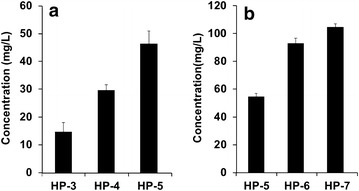
Effects of construct (**a**) or *E. coli* strain (**b**) on the production of *N*-(*p*-coumaroyl) phenethylamine

*N*-(*p*-Coumaroyl) phenethylamine is a conjugate of *p*-coumaric acid and phenethylamine. *p*-Coumaric acid is synthesized from tyrosine by TAL and phenethylamine is synthesized from phenylalanine by PDC. Therefore, the intracellular concentrations of both tyrosine and phenylalanine are critical for the final yield of *N*-(*p*-coumaroyl) phenethylamine. Tyrosine and phenylalanine are synthesized through the shikimate pathway. Two genes, *aroG* and *tyrA*, were overexpressed in order to increase the amount of tyrosine and phenylalanine. The gene *aroG*, which encodes 2-dehydro-3-deoxyphosphoheptonate aldolase, catalyzes the first reaction in the shikimate pathway. The gene *tyrA*, which encodes chorismate mutase/prephenate dehydrogenase, uses chorismate to make prephenate, which is used for the synthesis of phenylalanine and tyrosine. Both the AroG and TyrA proteins are inhibited by their final products (phenylalanine and tyrosine, repectively). Therefore, the feed back inhibition-free versions of *aroG* (*aroG*^*fbr*^) and *tyrA* (*tyrA*^* fbr*^) were used [27]. The production of *N*-(*p*-coumaroyl) phenethylamine was examined in three *E. coli* strains (HP-3, HP-4, and HP-5). HP-5 produced the highest amount of *N*-(*p*-coumaroyl) phenethylamine at approximately 46.1 mg/L, followed by HP-4 (29.6 mg/L), and HP-3 (14.6 mg/L) (Fig. 3a). Therefore, the tyrosine concentration in *E. coli* was directly related to the yield of *N*-(*p*-coumaroyl) phenethylamine. Next, we tested different *E. coli* mutants, which are expected to increase the production of tyrosine. The *tyrR* deletion mutant was the first mutant strain. TyrR is a transcription factor that is inhibited by tyrosine and phenylalanine. Deletion of *trpD*, which encodes the enzyme for the synthesis of tryptophan from chorismate, can result in higher substrate availability for the production of both tyrosine and phenylalanine. Therefore, two mutant strains, B-tryR (HP-6) and B-tyrR-typD (HP-7), both of which were transformed with the genes for *N*-(*p*-coumaroyl) phenethylamine, were used to examine the production of *N*-(*p*-coumaroyl) phenethylamine. Strain HP-7 produced 104.6 mg/L *N*-(*p*-coumaroyl) phenethylamine and HP-6 produced 92.9 mg/L (Fig. 3b). This result indicated that the final yield of *N*-(*p*-coumaroyl phenethylamine) was increased when more precursors for both tyrosine and phenylalanine are available.

**Fig. 4:**
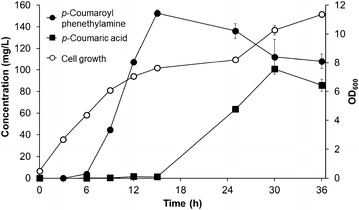
Production of *N*-(*p*-coumaroyl) phenethylamine using *E. coli* strain HP-7

Using the strain HP-7, the incubation temperature and the initial cell density were optimized. HP-7 was grown at18, 25, or 30 °C. The highest production of *N*-(*p*-coumaroyl) phenethylamine occurred in cells grown at 25 °C (106.1 mg/L), while the yields at 18 and 30 °C were approximately 90.8 and 88.5 mg/L, respectively. The optimal initial cell density was also examined. The cell density of HP-7 was adjusted with the culture medium to OD_600_ = 0.5, 1, or 1.5, and the production of *N*-(*p*-coumaroyl) phenethylamine was examined at the optimized temperature of 25 °C. The yield of *N*-(*p*-coumaroyl) phenethylamine was decreased from 136.1 mg/L at OD_600_ = 0.5, 106.1 mg/L at OD_600_ = 1.0 and 91.0 mg/L at OD_600_ = 1.5. Using the optimized incubation temperature and cell density of strain HP-7, the production of *N*-(*p*-coumaroyl) phenethylamine was monitored for 36 h. The production of *N*-(*p*-coumaroyl) phenethylamine was observed at 6 h and continued to increase until 15 h, when production was maximized. The yield of *N*-(*p*-coumaroyl) phenethylamine at 15 h was 152.5 mg/L. After 15 h, production was decreased (Fig. 4).

### Synthesis of *N*-(*p*-coumaroyl) tyramine in *E. coli*

**Fig. 5:**
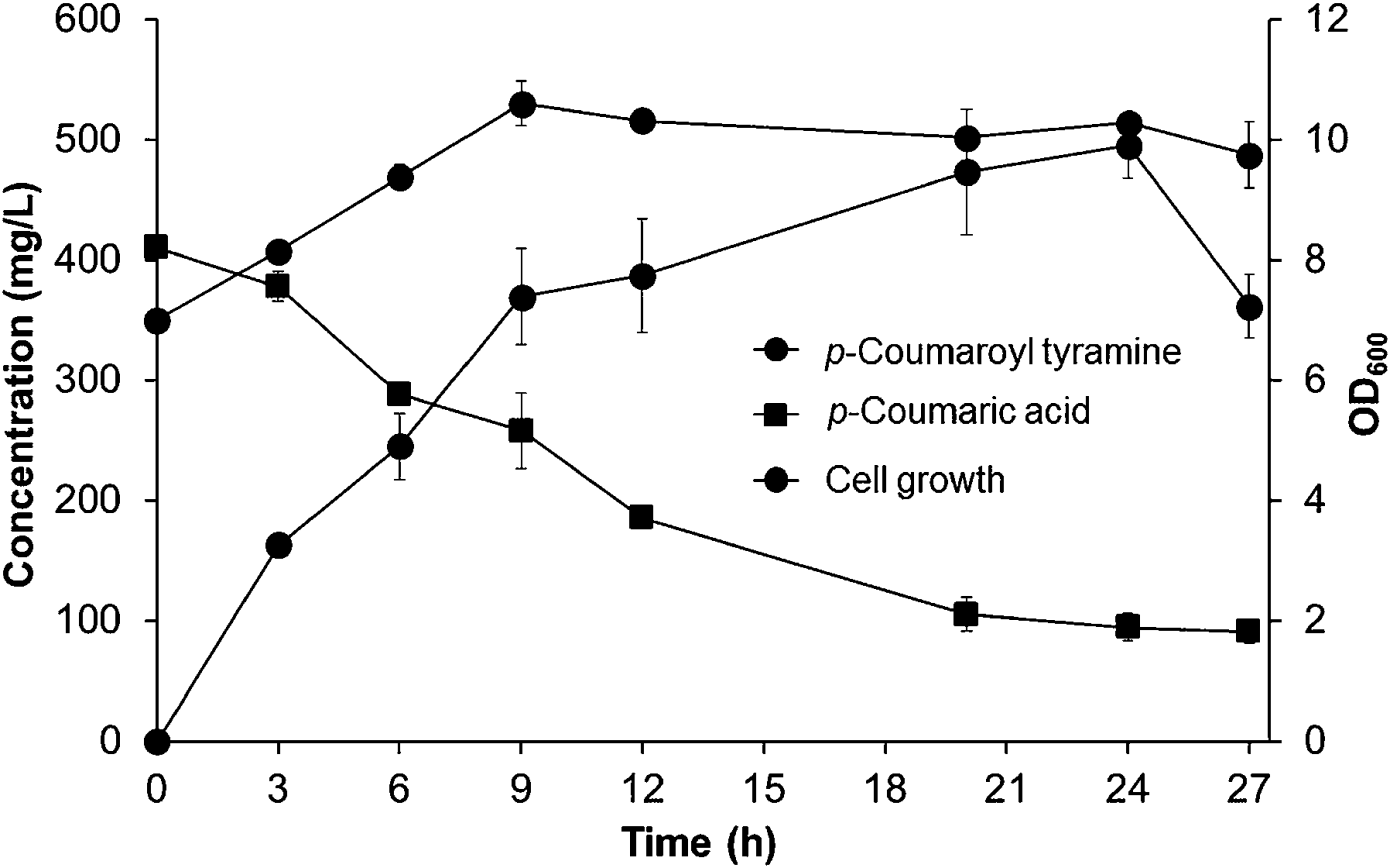
Production of *N*-(*p*-coumaroyl) tyramine by feeding *E. coli* strain HT-4 with *p*-coumaric acid

In order to synthesize *N*-(*p*-coumaroyl) tyramine by feeding with *p*-coumaric acid, an additional gene, *TDC* was introduced into HT-1. The resulting strain HT-2 was fed with *p*-coumaric acid and analysis of the reaction products using HPLC showed that *N*-(*p*-coumaroyl) tyramine was synthesized (data not shown). We also tested the optimal initial concentration of *p*-coumaric acid and found that the production of *N*-(*p*-coumaroyl) tyramine was increased up to 2.5 mM *p*-coumaric acid. Next, we tested the effect of tyrosine on the production of *N*-(*p*-coumaroyl) tyramine. Although *p*-coumaric acid was externally supplied to *E. coli*, tyramine needed to be synthesized from tyrosine. The strain HT-4, containing the feedback-inhibition free versions of *aroG* and *tyrA* (*aroG*^* fbr*^ and *tyrA*^* fbr*^) produced approximately 80.0 mg/L *N*-(*p*-coumaroyl) tyramine, which is more than was produced by strains HT-2 (48.2 mg/L) and HT-3 (58.9 mg/L). Using strain HT-4, we optimized the incubation temperature and initial cell concentration. The optimal incubation temperature and initial cell concentration were determined to be 30 °C and OD_600_ = 7, respectively. Using HT-4, approximately 495.4 mg/L *N*-(*p*-coumaroyl) tyramine was synthesized after 24 h of incubation, after which the production decreased (Fig. 5).

**Fig. 6:**
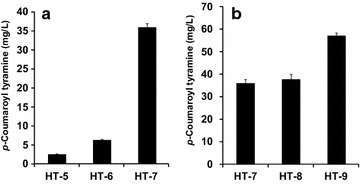
Effect of different gene combinations (**a**) and *E. coli* strains (**b**) on the production of *N*-(*p*-coumaroyl) tyramine from glucose in *E. coli*

Next, we synthesized *N*-(*p*-coumaroyl) tyramine from tyrosine. For this, the intracellular concentration of tyrosine is critical. Therefore, we used the same strategies as used for the synthesis of *N*-(*p*-coumaroyl) phenethylamine in order to supply more tyrosine. We examined the effects of different constructs and *E. coli* mutants on the production of *N*-(*p*-coumaroyl) tyramine. As expected, the strain HT-7 (36.0 mg/L) produced more tyrosine than the other strains did (HT-6, 6.3 mg/L and HP-5, 2.5 mg/L) (Fig. 6a). Therefore, the tyrosine concentration in *E. coli* was directly related to the yield of *N*-(*p*-coumaroyl) tyramine. Also, the *E. coli* mutant, BtyrR-trpD (HT-9, 57.1 mg/L) produced more *N*-(*p*-coumaroyl) tyramine than BtyrR-pheA (HT-8, 37.8 mg/L). Again, the intracellular tyrosine concentration was directly related to the final yield of *N*-(*p*-coumaroyl) tyramine (Fig. 6b).

In order to optimize the production of *N*-(*p*-coumaroyl) tyramine using the strain HT-9, the incubation temperature and initial cell density were optimized. The best temperature was determined to be 30 °C (57.1 mg/L) followed by 25 °C (52.5 mg/L), 37 °C (39.2 mg/L), and 18 °C (22.0 mg/L).

Using the strain HT-9, the incubation temperature and the initial cell density were optimized. HT-9 was grown at 18, 25, 30, or 37 °C. The highest production of *p*-coumaroyl tyramine occurred in cells grown at 30 °C (57.06 mg/L). The yields at 18, 25 and 37 °C were approximately 21.98, 52.47 and 39.16 mg/L, respectively. The optimal initial cell density was also examined. The production of *N*-(*p*-coumaroyl) tyramine increased from OD_600_ = 0.5 to OD_600_ = 2 but decreased at OD_600_ = 3.

**Fig. 7:**
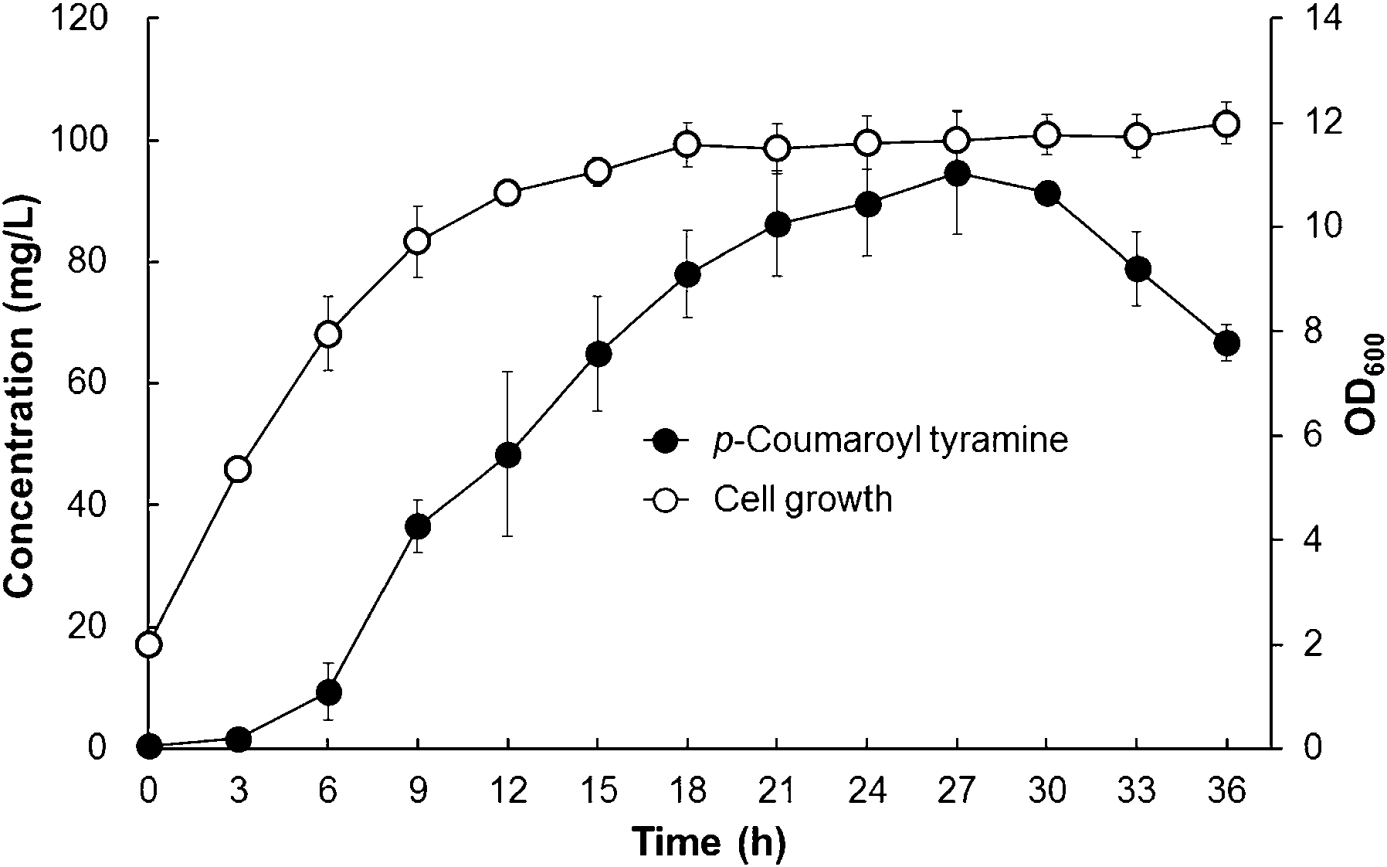
Production of *N*-(*p*-coumaroyl) tyramine using *E. coli* strain HT-9

The production of *N*-(*p*-coumaroyl) tyramine was monitored for 36 h with strain HT-9. The production of *N*-(*p*-coumaroyl) tyramine was observed at 3 h and continued to increase until 27 h, at which time the production of *N*-(*p*-coumaroyl) tyramine was maximized. The yield of *N*-(*p*-coumaroyl) tyramine at 27 h was 94.7 mg/L. After 27 h, the production decreased (Fig. 7).

## Discussion

Five *N*-HC phenethylamines and eight *N*-HC tyramines from 12 HCAs and corresponding amines (phenethylamine and tyramine) were synthesized. Two enzymatic reactions are required to synthesize *N*-HC phenethylamines or *N*-HC tyramines in *E. coli*. The first step is the activation of HC by 4CL which results in the formation of HC-CoA. The second step is the conjugation of HC-CoA to phenethylamine or tyramine, which is catalyzed by either SHT or THT. Based on the results of HT-1 biotransformation, Os4CL used in this study could use at least eight HCAs as substrates (*p*-coumaric acid, *m*-coumaric acid, *o*-coumaric acid, caffeic acid, ferulic acid, cinnamic acid, 3-methoxycinnamic acid, and 4-methoxycinnamic acid). However, HCAs having two methoxy groups did not yield any reaction products. It is not clear whether this result is due to the failure of Os4CL to convert HCA into HC-CoA or due to the formation of conjugates between HC-CoA and tyramine or phenethylamine by THT or SHT. These results also suggested that THT, which was used for the formation of *N*-HC tyramines, had broader acyl donor specificity than did SHT, which was used for the synthesis of *N*-HC phenethylamines.

It is not clear whether the different rates of *N*-HC phenethylamines and *N*-HC tyramines formation observed can be attributed to the first step or the second step. It is, however, clear that Os4CL used *p*-coumaric acid or caffeic acid more efficiently than it used ferulic acid [28]. In the formation of *N*-HC tyramine, *N*-feruloyl tyramine was synthesized more effectively than *N*-(*p*-coumaroyl) tyramine or *N*-caffeoyl tyramine. These observations suggest that the second reaction catalyzed by THT determines the rate of product formation.

We synthesized *N*-(*p*-coumaroyl) phenethylamines and *N*-(*p*-coumaroyl) tyramines using three different approaches. Firstly, HC and either phenethylamine or tyramine were used as substrates. Two genes, *4CL* and either *SHT* for *N*-(*p*-coumaroyl) phenethylamine biosynthesis or *THT* for *N*-(*p*-coumaroyl) tyramine biosynthesis, were transformed into *E. coli* and various HC-amines were successfully synthesized. Secondly, HC and either phenylalanine or tyrosine were used. For this, an additional gene, either *PDC* or *TDC* was introduced to convert phenylalanine or tyrosine into phenethylamine or tyramine, respectively. Thirdly, we synthesized *N*-(*p*-coumaroyl) phenethylamine and *N*-(*p*-coumaroyl) tyramine from amino acids (phenylalanine and tyrosine, respectively) by introducing *TAL* into *E. coli*. In these approaches, the yield of the major product was much higher than that of byproduct(s). It is likely that it is related to the specificity of the enzymes employed in this study. During screening of HCTs using biotransformation, we observed that both SHT and THT used both phenethylamine and tyramine (Fig. 2), although SHT showed a preference for phenethylamine and THT showed a preference for tyramine. Previous in vitro kinetic data have also shown that SHT has a higher preference for phenethylamine than for tyramine [25]. This is one reason that only a small amount of byproduct is observed. Moreover, the amino acid decarboxylases (PDC and TDC) used in this study showed high substrate specificities. PDC from *Pseudomonas putida* showed higher substrate specificity for phenylalanine than for tyrosine [29] and TDC from *P. somniferum* showed activity for tyrosine but not for phenylalanine [20]. Furthermore, the TAL we used was previously found to show higher substrate specificity for tyrosine [30] than for phenylalanine. Therefore, byproducts were rarely synthesized. Taken together, using specific enzymes for each reaction step contributed to the synthesis of a specific product with only the formation of a small amount of byproduct(s).

## Conclusions

HC-amine conjugates such as *N*-HC tyramines and *N*-HC phenethylamines have been considered as potential starting materials for development of antiviral and anticancer drus. We synthesized *N*-HC tyramines and *N*-HC phenethylamines using *E. coli* harboring *4CL* and either *THT* or *SHT*. Approximately 101.9 mg/L *N*-(*p*-coumaroyl) phenethylamine and 495.4 mg/L *N*-(*p*-coumaroyl) tyramine were synthesized from *p*-coumaric acid using *E. coli* harboring an additional gene either *PDC* or *TDC*. Finally, we synthesized 152.5 mg/L *N*-(*p*-coumaroyl) phenethylamine and 94.7 mg/L *N*-(*p*-coumaroyl) tyramine from glucose by engineering the shikimate pathway of *E. coli* and reconstructing the pathway toward synthesis of *N*-(*p*-coumaroyl) phenethylamine and *N*-(*p*-coumaroyl) tyramine. Productivity was further improved by optimizing the cell concentration and incubation temperature.

## Methods

### Constructs and *E. coli* strains

*Os4CL* was previously cloned in our lab [28] and was subcloned into the *Eco*RI/*Not*I sites of pCDFDuet1 (pC-Os4CL). HC-CoA: serotonin *N*-(HC) transferase (*CaSHT*) from *Capsicum annuum* (GenBank accession number AF329463.1) for the synthesis of *N*-(*p*-coumaroyl) phenethylamine was cloned using reverse transcription-polymerase chain reaction (RT-PCR). cDNA was isolated from the leave of *C. annuum*. PCR was carried out using primers, 5′-ATGATATCGATGGCTTCTGCTCCTCAACCACCAA-3′ (*Eco*RV site is underlined.) as a forward primer and 5′-ATCTCGAGCTAACAGCTTCCTGCACCATTTTTCT-3′ (*Xho*I site is underlined.) as a reverse primer. The resulting PCR product was subcloned into the *Eco*RV/*Xho*I sites of pC-Os4CL and named pC-Os4CL-SHT.

*PDC* from *P. putida* (GenBank accession number BK006920) was amplified using PCR using genomic DNA as a template using 5′-ATCATATGGTGACCCCCGAACAATTCCGCC-3′ (*Nde*I site is underlined.) as a forward primer and 5′-AACTCGAGTCAGCCCTTGATCACGTCCTGC-3′ (*Xho*I site is underlined.) as a reverse primer. The resulting PCR product was subcloned into the *Nde*I/*Xho*I sites of pET-Duet1 (Novagene, Madison, WI, USA) and the construct was named pE-PDC. *TDC* from *Papaver somniferum* was synthesized after codon optimization for *E. coli* based on the published sequence (GenBank U08598.1) and subcloned into *Bam*HI/*Hin*dIII sites of pCDF. The construct formed was named pC-TDC. *Os4CL* was amplified with a forward primer containing T7 promoter sequence and *Hin*dIII site and a reverse primer containing a *Not*I site. The resulting PCR product was subcloned into the *Hin*dIII/*Not*I sites of pC-TDC and the construct was named pC-TDC-Os4CL. Tyramine *N*-HC transferase (THT) from *C. annuum* (GenBank accession number AY819700.1) was cloned using RT-PCR. The resulting PCR product was subcloned into the *Nde*I/*Eco*RV sites of pC-TDC-Os4CL and the construct was named pC-TDC-Os4CL-THT.

pA-SeTAL, pA-aroG-SeTAL-tyrA, and pA-aroG^fbr^-SeTAL-tyrA^fbr^ were cloned previously [31].

### Synthesis of *N*-HC phenethylamines and *N*-HC tyramines

HCAs including *p*-coumaric acid, *m*-coumaric acid, *o*-coumaric acid, caffeic acid, ferulic acid, cinnamic acid, 3-methoxycinnamic acid, 4-methoxycinnamic acid, 3,4-dimethoxycinnamic acid, 2,4-dimethoxycinnamic acid, sinapic acid, and 3,4,5-trimethoxycinnamic acid were purchased from Sigma-Aldrich (St. Louis, MO, USA). Preference of HCA for the synthesis of *N*-HC phenethylamines or *N*-HC tyramines was tested as follows. *E. coli* strains HP-1 and (Table 1) for the synthesis of *N*-HC phenethylamines and *N*-HC tyramines, respectively were grown at 37 °C for 18 h in LB medium containing 50 μg/mL spectinomycin. The culture was inoculated into a fresh new LB medium containing 50 μg/mL spectinomycin. Cells were grown until OD_600_ = 0.8, at which point 1 mM isopropyl β-d-1-thiogalactopyranoside (IPTG) was added, and continued to grow at 18 °C for a further 24 h. The cells were harvested and resuspended in M9 medium containing 2 % (w/v) glucose, 1 % (w/v) yeast extract, either 500 μM HCA (for the synthesis of *N*-HC phenethylamines) or 1 mM HCA (for the synthesis of HC-tyramine), 100 μg/ml antibiotic(s), and 1 mM IPTG. Fourteen HC derivatives (*p*-coumaric acid, *m*-coumaric acid, *o*-coumaric acid, caffeic acid, ferulic acid, cinnamic acid, 3-methoxycinnamic acid, 4-methoxycinnamic acid, 3,4-dimethoxycinnamic acid, 2,4-dimethoxycinnamic acid, sinapic acid, and 3,4,5-trimethoxycinnamic acid) were used, individually. The cultures were incubated at 30 °C for 3 h and then extracted with ethylacetate. After drying the aqueous layer and dissolving it in dimethylsulfoxide (DMSO), the product was analyzed by HPLC. The relative *N*-HC phenethylamine or *N*-HC tyramine formation rates were calculated relative to that of the best substrate, which was denoted as 100 %.

The metabolites were analyzed using an UltiMate 3000 HPLC (Thermo Scientific, Waltham, MA, USA) equipped with a photo diode array (PDA) detector and a C18 reversed-phase column (4.60 × 250 mm, 3.5 μm particle size, Varian, Palo Alto, CA, USA). The mobile phase consisted of 0.1 % (v/v) formic acid in water and acetonitrile. The program was as follows: 20 % (v/v) acetonitrile at 0 min, 60 % at 8 min, 90 % at 12 min, 90 % at 15 min, 20 % at 15.1 min, 20 % at 20 min. The flow rate was 1 ml/min and UV absorbance was monitored at 278 and 310 nm.

Mass spectrometry (MS) was carried out as described previously [32]. The structures of *N*-(*p*-coumaroyl) phenethylamine and *N*-(*p*-coumaroyl) tyramine were determined using nuclear NMR [33]. The NMR data were as follows; *N*-(*p*-coumaroyl) phenethylamine: ^1^H NMR (400 MHz, DMSO-*d*_*6*_) δ 7.39 (d, *J* = 8.6 Hz, 2H), 7.34 (d, *J* = 15.7 Hz, 1H), 7.29 (m, 2H), 7.22 (d, *J* = 7.97, 2H), 7.20 (dd, *J* = 16.4, 7.5 Hz, 1H), 6.80 (d, *J* = 8.6 Hz, 2H), 6.42 (d, *J* = 15.7 Hz, 1H), 3.41 (m, 2H), 2.78 (t, *J* = 3.5 Hz, 2H); ^13^C NMR (100 MHz, DMSO-*d*_*6*_) δ 165.3, 158.8, 139.5, 138.6, 129.2, 128.6, 128.3, 126.0, 125.9, 118.6, 115.7, 40.3, 35.2.

*N*-*(p*-Coumaroyl) tyramine: ^1^H NMR (400 MHz, DMSO-*d*_*6*_) δ 8.00 (s, NH), 7.38 (m, 2H), 7.31 (d, *J* = 15.7 Hz, 1H), 7.00 (m, 2H), 6.79 (m, 2H), 6.68 (m, 2H), 6.40 (d, *J* = 15.7 Hz, 1H), 3.32 (dd, *J* = 7.4, 6.3 Hz, 2H), 2.64 (t, *J* = 7.4 Hz, 2H); ^13^C NMR (100 MHz, DMSO-*d*_*6*_) δ 165.3, 158.8, 155.6, 138.5, 129.5, 129.4, 129.1, 125.9, 118.7, 115.7, 115.1, 40.6, 34.4.

*N*-(*p*-Coumaroyl) phenethylamine and *N*-*(p*-coumaroyl) tyramine were collected and used as standards for the calculation of the production of other *N*-HC phenethylamines and *N*-HC tyramines, respectively.

### Total synthesis of *N*-(*p*-coumaroyl) phenethylamine and *N*-(*p*-coumaroyl) tyramine

Overnight cultures of *E. coli* strains, HTs or HPs (Table 1) were inoculated into 15 ml of fresh LB medium containing appropriate antibiotics and were cultured to OD_600_ = 1. Cells were harvested by centrifugation and cell density was adjusted to OD_600_ = 2 with 10 mL of M9 medium containing 2 % (w/v) glucose, 1 % (w/v) yeast extract, 50 μg/mL antibiotics, and 1 mM IPTG in a 100 mL flask. The cells were grown at 30 °C with shaking at 180 rpm for 24 h. To analyze product formation, cell growth was monitored by determining the absorbance at 600 nm. Culture supernatants were collected, extracted twice with an equal volume of ethyl acetate, and then dried under vacuum. The dried samples were dissolved in DMSO and analyzed using HPLC.

The mean and the standard deviation (SD) were calculated from triplicate experiments. Analysis of variance (ANOVA) was carried out using Tukey’s method, with significance at a P value of 0.01, using Microsoft Excel.
